# CRISPR/Cas9-mediated activation of *NR5A1* steers female human embryonic stem cell-derived bipotential gonadal-like cells towards a steroidogenic cell fate

**DOI:** 10.1186/s13048-023-01264-5

**Published:** 2023-09-20

**Authors:** Laura Danti, Karolina Lundin, Kirsi Sepponen, Dawit A. Yohannes, Juha Kere, Timo Tuuri, Juha S. Tapanainen

**Affiliations:** 1 Department of Obstetrics and Gynecology, University of Helsinki and Helsinki University Hospital, Helsinki, 00290 Finland; 2 Research Programs Unit, Translational Immunology & Department of Medical and Clinical Genetics, University of Helsinki, Helsinki, 00290 Finland; 3 Folkhälsan Research Centre, Stem Cells and Metabolism Research Program, University of Helsinki, Helsinki, 00290 Finland; 4 Department of Biosciences and Nutrition, Karolinska Institutet, Huddinge, 14183 Sweden; 5 Department of Obstetrics and Gynecology, HFR – Cantonal Hospital of Fribourg and University of Fribourg, Fribourg, 1708 Switzerland

**Keywords:** CRISPR, NR5A1, SF-1, Orphan nuclear receptor, Ovary, Testis, Steroidogenesis, Gonadal development, Stem cells

## Abstract

The nuclear receptor subfamily 5 group A member 1 (*NR5A1*), encoding steroidogenic factor 1 (SF-1), has been identified as a critical factor in gonadal development in animal studies. A previous study of ours suggested that upregulation of *NR5A1* during early gonadal differentiation in male (46,XY) human pluripotent stem cells steers the cells into a more mature gonadal cell type. However, the detailed role of *NR5A1* in female gonadal differentiation has yet to be determined. In this study, by combining the processes of gonadal differentiation and conditional gene activation, we show that *NR5A1* induction predominantly upregulates the female gonadal marker inhibin subunit α (*INHA)* and steroidogenic markers steroidogenic acute regulatory protein (*STAR*), cytochrome P450 family 11 subfamily A member 1 (*CYP11A1*), cytochrome P450 family 17 subfamily A member 1 (*CYP17A1*), hydroxy-delta-5-steroid dehydrogenase (*HSD3B2*) and hydroxysteroid 17-beta dehydrogenase 1 (HSD17B1). In contrast, *NR5A1* induction did not seem to affect the bipotential gonadal markers gata binding protein 4 (*GATA4*) and Wilms’ tumour suppressor 1 *(WT1*) nor the female gonadal markers r-spondin 1 (*RSPO1*) and wnt family member 4 (*WNT4*). Differentially expressed genes were highly associated with adrenal and ovarian steroidogenesis pathways. Moreover, time-series analysis revealed different dynamic changes between male and female induced samples, where continuously upregulated genes in female gonadal differentiation were mostly associated with adrenal steroidogenesis. Thus, in contrast to male gonadal differentiation, *NR5A1* is necessary but not sufficient to steer human embryonic stem cell (hESC)-derived bipotential gonadal-like cells towards a more mature somatic, female cell fate. Instead, it seems to direct bipotential gonadal-like cells more towards a steroidogenic-like cell population. The information obtained in this study helps in elucidating the role of *NR5A1* in gonadal differentiation of a female stem cell line.

**Supplementary Information:**

The online version contains supplementary material available at 10.1186/s13048-023-01264-5.

## Background

In humans, the gonads develop from the genital ridges, which are paired structures on the ventromedial surface of the mesonephros. Genital ridges form as a result of thickening of intermediate mesoderm and proliferation of overlaying coelomic epithelium during the latter part of the fifth week of development [1, 2]. At this stage the gonads are morphologically indistinguishable in both sexes and called bipotential gonads [3]. Sex differentiation occurs around the seventh week of development and is dependent on the chromosomal composition. A genetically male (XY) embryo has the sex-determining region on the Y chromosome (*SRY*), which initiates testicular development [4–6]. In the absence of *SRY*, thus in a genetically female (XX) embryo, ovarian development is initiated by the female-specific markers wnt family member 4 (*WNT4)* [7, 8], r-spondin 1 (*RSPO1)* and forkhead box protein L2 (*FOXL2*) [9–12].

The bipotential gonad expresses genes essential for gonadal development, including empty spiracles homeobox 2 (*EMX2*), lim homeobox 9 (*LHX9*), Wilms’ tumour suppressor 1 (*WT1*), gata binding protein 4 (*GATA4*) and nuclear receptor subfamily 5 group A member 1 (*NR5A1*) [13]. The *NR5A1* gene encodes an orphan nuclear receptor, steroidogenic factor 1 (SF-1), which is a member of the nuclear receptor superfamily initially identified as a master-regulator of steroidogenic enzymes in adrenocortical cells [14–16].

*NR5A1* plays an important role in the development and function of steroidogenic tissues, gonads and adrenal glands [17–19]. *NR5A1* knock-out (KO) mice completely lack gonads and adrenal glands, present with female external genitalia irrespective of genetic sex and die within eight days after birth [17, 20, 21]. However, gonad-specific KO completely bypasses the lethality of *NR5A1*-null mice in both sexes [22, 23]. Testis-specific KO of *NR5A1* causes abnormalities in the testes from very early developmental stages. The testes of adult KO mice were found to be at the level of the bladder and hypoplastic, partly due to impaired spermatogenesis [22]. In the case of ovary-specific KO of *NR5A1*, wild-type and KO ovaries were indistinguishable from each other during embryogenesis and at birth. However, adult females were found to be sterile, most likely due to defects in steroidogenesis [22, 23].

In humans, there are three recorded homozygous *NR5A1* mutations (R92Q, D293N and R103Q). In 46,XY patients the symptoms range from primary adrenal insufficiency to complete gonadal dysgenesis and differences in sex development (DSD). Homozygous *NR5A1* mutations have been found in 46,XX patients with primary adrenal insufficiency and primary ovarian failure [24]. Heterozygous *NR5A1* mutations may rarely cause isolated adrenal insufficiency, but are usually accompanied by gonadal dysfunction and dysgenesis [25].

In one of our previous studies we showed that *NR5A1* seems to direct male (XY) human induced pluripotent stem cells (hiPSC), differentiated into bipotential gonadal-like cells, towards more mature somatic gonadal-like cells [26]. In the present study, we conditionally activated *NR5A1* in a female human embryonic stem cell (hESC) line during gonadal differentiation to study the role of *NR5A1* in female gonadal differentiation. We showed that activating *NR5A1* in female hESCs seems to steer bipotential gonadal-like cells towards a steroidogenic-like cell population instead of a more mature gonadal cell type, as is the case in gonadal differentiation of the male line.

## Results

### Bipotential and female gonadal markers are upregulated during gonadal differentiation

**Fig. 1:**
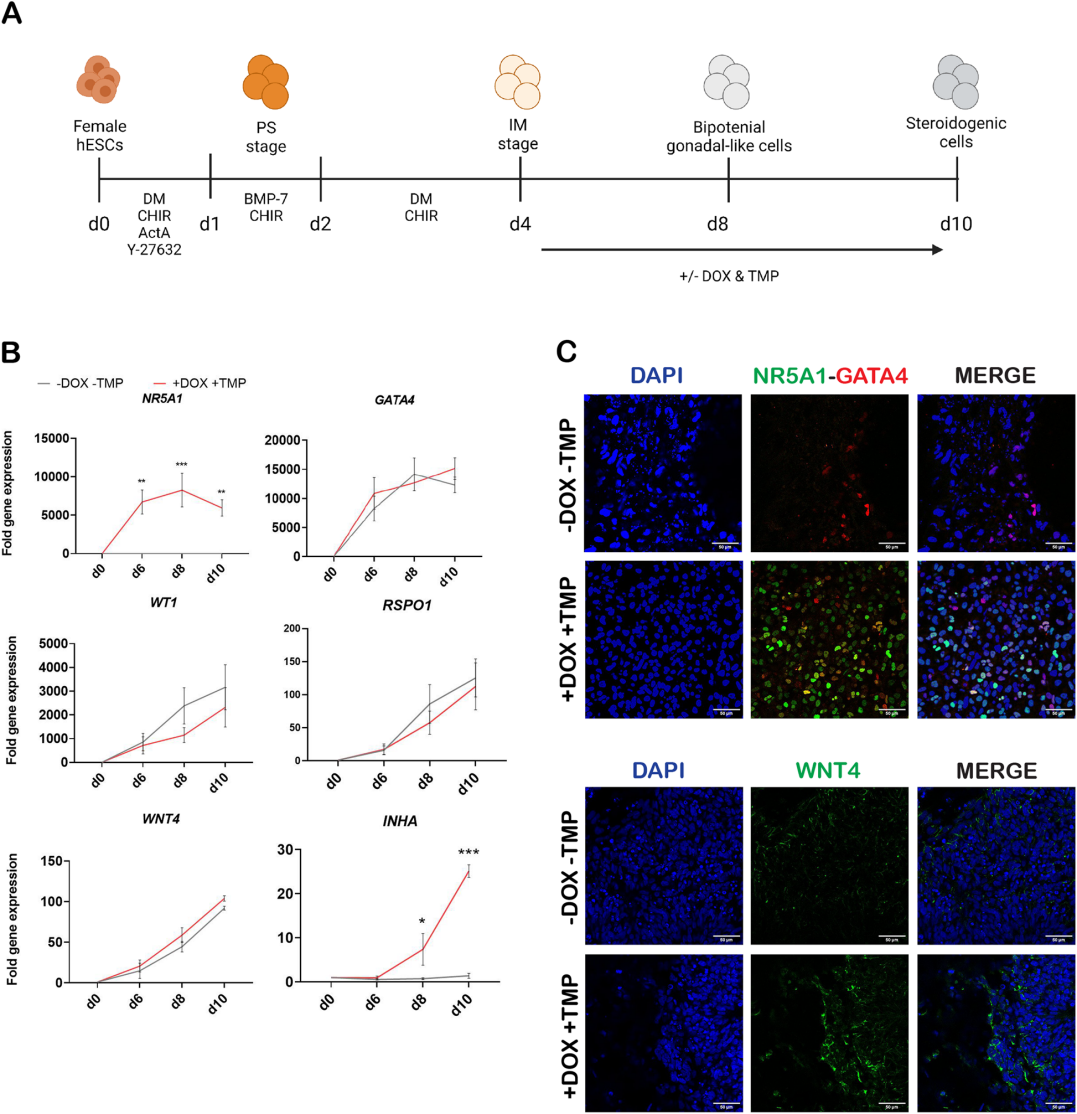
*NR5A1* induction has a minimal effect on the upregulation of bipotential gonadal and female markers. **A** A schematic representation of the gonadal differentiation protocol from hESCs to steroidogenic-like cells with the matching developmental stages and administered growth factors, inhibitors, and small molecules. **B** RT-qPCR analysis of markers associated with gonadal development showed that *NR5A1* induction was successful. Bipotential gonadal markers (*NR5A1*, *GATA4*,* WT1*) were upregulated by day 8, female gonadal markers (*RSPO1*,* WNT4*) were upregulated by day 10 as a result of gonadal differentiation even without *NR5A1* induction and *INHA *was significantly upregulated after *NR5A1* induction. Data are reported as mean ± SEM, *n*=3 biological replicates. Two-way ANOVA; 0.12 (ns), 0.033 (*), 0.002 (**), < 0.001 (***). **C** Confocal images of immunofluorescence staining showed expression of bipotential markers NR5A1 and GATA4, and the female gonadal marker WNT4 in non-induced (-DOX -TMP) and induced (+DOX +TMP) conditions at day 10 of gonadal differentiation. Images were taken with a ×40 objective. Scale bars 50 µm. ActA, activin A; BMP, bone morphogenetic protein; CHIR, CHIR-99021; DM, dorsomorphin; hESCs, human embryonic stem cells; IM, intermediate mesoderm; PS, primitive streak; d, day of differentiation; DOX, doxycycline hyclate; TMP, trimethoprim

To study the role of *NR5A1* in human embryonic development, a dual inducible *NR5A1* activation line was established using the CRISPR/Cas9 genome editing technique, involving H9-*NR5A1*-DDdCas9VP192 hESC clonal line 10. This cell line was subjected to a gonadal differentiation protocol (Fig. 1A), based on a previous protocol established by Sepponen et al., 2017 [27]. *NR5A1* was induced by the simultaneous administration of doxycycline hyclate (DOX) and trimethoprim (TMP) on day 4 (at the intermediate mesoderm stage) until day 10 of differentiation. RT-qPCR analyses showed that in the induced state (+ DOX + TMP), *NR5A1* was significantly upregulated at days 6, 8 and 10 of differentiation, whilst being completely absent in the non-induced state (-DOX -TMP) (Fig. 1B). During the differentiation process, the bipotential markers *GATA4* and *WT1* were upregulated by day 8 of differentiation, but *NR5A1* induction did not seem to affect their gene expression levels. A similar trend was seen for the female gonadal markers *RSPO1* and *WNT4* by day 10 of gonadal differentiation. However, the ovarian marker inhibin subunit α (*INHA*) was significantly upregulated on days 8 and 10 of gonadal differentiation by *NR5A1* induction (Fig. 1B).

Immunofluorescence co-staining of NR5A1 and GATA4 at day 10 of differentiation showed the presence of NR5A1 in the induced state, whereas it was completely undetectable in the non-induced state (Fig. 1C). GATA4, on the other hand, was present in both conditions at similar staining intensities. Likewise, WNT4 was expressed in both conditions with a similar intensity in the non-induced and induced state (Fig. 1C).

### *NR5A1* induces steroidogenesis during gonadal differentiation

**Fig. 2:**
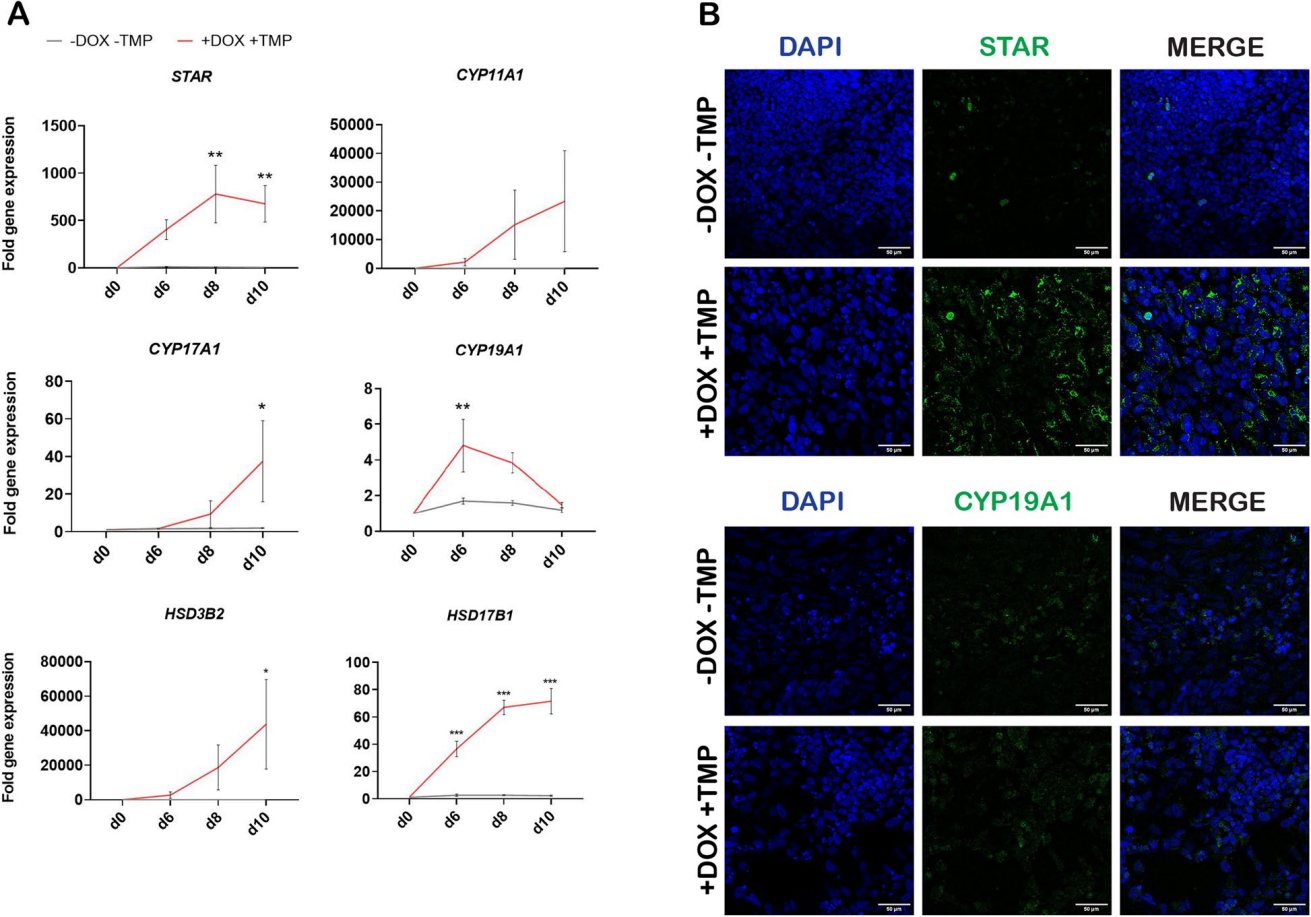
*NR5A1* induction upregulates steroidogenesis markers but downregulates the aromatase enzyme *CYP19A1*. **A **RT-qPCR analysis of markers associated with steroidogenesis showed that *NR5A1* induction upregulated the markers *STAR*, *CYP11A1*, *CYP17A1*, *HSD3B2* and *HSD17B1*, but downregulated the aromatase enzyme *CYP19A1*. Data are reported as mean ± SEM, *n*=3 biological replicates. Two-way ANOVA; 0.12 (ns), 0.033 (*), 0.002 (**), < 0.001 (***). **B **Confocal images of immunofluorescence staining showed expression of steroidogenic markers STAR and CYP19A1 in non-induced (-DOX -TMP) and induced (+DOX +TMP) conditions at day 10 of gonadal differentiation. Images were taken with a ×40 objective. Scale bars 50 µm. d, day of differentiation; DOX, doxycycline hyclate; TMP, trimethoprim

*NR5A1* induction resulted in upregulation of several steroidogenesis markers. RT-qPCR analyses showed that levels of steroidogenic acute regulatory protein (STAR) mRNA were increased on day 6 and significantly increased on days 8 and 10 of differentiation in the induced state, but no STAR mRNA expression was detectable in the non-induced state (Fig. 2A). Similarly, cytochrome P450 family 11 subfamily A member 1 (*CYP11A1*) and cytochrome P450 family 17 subfamily A member 1 (*CYP17A1*) mRNA expression levels were upregulated on days 6, 8 and 10 upon *NR5A1* induction, with the increase in *CYP17A1* being significant on day 10. Interestingly, cytochrome P450 family 19 subfamily A member 1 (*CYP19A1*) levels decreased over the course of gonadal differentiation and upon *NR5A1* induction, with the decrease on day 6 being statistically significant. Hydroxy-delta-5-steroid dehydrogenase (*HSD3B2*) showed a continuous upregulation upon *NR5A1* induction, with the increase in gene expression being significant on day 10. Lastly, hydroxysteroid 17-beta dehydrogenase 1 (HSD17B1) showed a similar trend with the upregulation being significant across all timepoints (Fig. 2A).

Immunofluorescence staining at day 10 of gonadal differentiation showed minimal presence of STAR in the non-induced state, whereas its staining intensity was increased in the induced condition. In contrast, there was little difference in CYP19A1 expression between the two conditions (Fig. 2B).

### *NR5A1* induces transcriptional changes during differentiation of bipotential gonadal-like cells

To further study the effect of *NR5A1* expression during differentiation, we performed bulk RNA sequencing (RNA-seq) analyses on *NR5A1*-induced (IND, +DOX + TMP) and non-induced (CTRL, -DOX -TMP) samples taken on days 4, 6, 8 and 10 of differentiation. Principal Component Analysis (PCA) of the top 500 most variable genes showed clear separate clustering of the induced and non-induced samples, as well as clear divergence between the different days of differentiation (Suppl. Figure 1 A). According to the PCA results, in gonadal differentiation of the female line, about 52% of the variance observed between the samples could be attributed to the differentiation protocol itself (Principal Component 1, PC1) and 34% of the variance was due to *NR5A1* induction (Principal Component 2, PC2) (Suppl. Figure 1 A).

Furthermore, we performed pairwise differential expression (DE) comparisons between samples at different timepoints and between induced (IND, +DOX + TMP) and non-induced (CTRL, -DOX-TMP) conditions at a specific timepoint during differentiation. Additionally, we compared the number of differentially expressed genes (DEGs) (coding and non-coding, upregulated and downregulated) in the female line and the male line, the latter having been previously reported [26] (Suppl. Figure 1B). Two main differences were found between the female and male lines undergoing gonadal differentiation as regards DEGs. Firstly, in the female line the highest number of DEGs was found at day 6 of differentiation (3,496), whilst in the male line this was at day 10 of differentiation (5,721). Secondly, there were more upregulated DEGs at each timepoint in the female line subjected to gonadal differentiation, whilst in the male line at each timepoint there were more downregulated DEGs. However, one similarity was found: at each timepoint in both the female and male lines the coding DEGs dominated over the non-coding DEGs in conditions of both upregulated and downregulated DEGs (Suppl. Figure 1B).

**Fig. 3:**
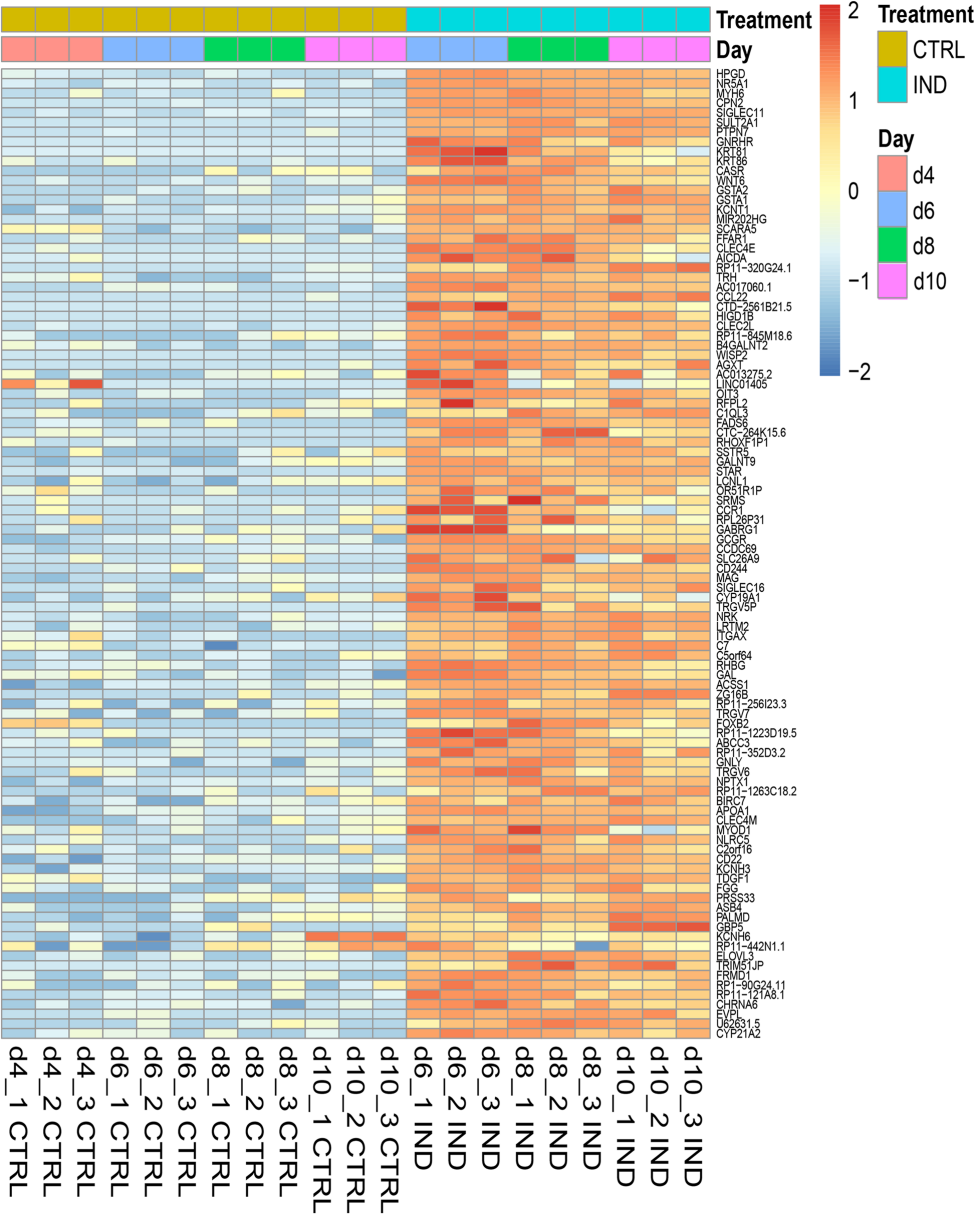
Expression pattern of the top 100 upregulated DE genes at day 6 of differentiation upon *NR5A1* induction

Next, we plotted a heatmap to represent the expression profile of the top 100 upregulated DEGs with the highest fold change at day 6 of gonadal differentiation in the female line. *NR5A1* induction was followed by clear upregulation of this set of genes at all timepoints studied (Fig. 3). We selected several genes from this list that were known to be associated with female somatic gonadal cells or steroidogenesis and performed RT-qPCR analyses to validate the RNA-seq results (Suppl. Figure 2 A). The results showed that indeed 15-hydroxyprostaglandin dehydrogenase (*HPGD*), glutathione S-transferase A1 (*GSTA1*), glutathione S-transferase A2 (*GSTA2*), wnt family member 6 (*WNT6*), beta-1,4-N-acetyl-galactosaminyltransferase 2 (*B4GALNT2*) and gonadotropin releasing hormone receptor (*GNRHR*) mRNA expression levels were induced upon *NR5A1* induction with DOX and TMP (Suppl. Figure 2 A).

The heat map shows comparison of non-induced (CTRL, -DOX-TMP) and induced (IND, +DOX + TMP) RNA-sequenced samples across all timepoints. The top 100 DEGs at day 6 of differentiation are displayed and each rectangle represents the expression of a specific gene within a technical replicate. The intensity of gene expression is indicated by a colour scale based on row z-scores (red, highest expression levels; blue, lowest expression levels). d, day.

To further understand the effect of *NR5A1* induction on the differentiation of bipotential gonadal-like cells in the female line, we performed Kyoto Encyclopedia of Genes and Genomes (KEGG) enrichment analyses on the DEGs at days 6, 8 and 10 of differentiation using the online ENRICHR tool (Suppl. Figure 2B). ‘Cortisol synthesis and secretion’ was the dominant associated pathway on days 6, 8 and 10 of differentiation. ‘Aldosterone synthesis and secretion’ was the second most associated pathway with DEGs on day 6, whilst on day 8 this was ‘cell adhesion molecules’, and ‘neuroactive ligand-receptor interaction’ on day 10. Across all three timepoints, the KEGG pathway ‘ovarian steroidogenesis’ was also associated with upregulated DEGs (Suppl. Figure 2B).

Lastly, we looked at the expression profiles of genes known to be associated with ovarian, testicular or adrenal steroidogenesis from previously published papers by Mamsen et al. [28], del Valle et al. [29] and Lecluze et al. [30]. Most of the genes associated with steroidogenesis were clearly upregulated in our datasets upon *NR5A1* activation (Suppl. Figure 3).

#### Different dynamic changes in expression pattern were induced by *NR5A1* in the female and male human pluripotent stem cell (hPSC) lines

In normal gonadal development the future fate of bipotential gonadal cells is largely dependent on the presence or absence of SRY. Moreover, ovaries and testes are inherently different organs that develop differently. Therefore, the sequencing data from gonadal differentiation of the female line was further compared with the previously published sequencing data from gonadal differentiation of the male line [26] in both control and induced conditions. We performed time-series analysis on the *NR5A1*-induced DEGs to identify the genes that presented dynamic changes in their expression paths during differentiation. These paths were grouped first according to their expression in either female or male samples and induced and non-induced conditions. Secondly, they were grouped according to their direction of expression at each timepoint. For example, genes showing continuous upregulation over the timepoints (d4 > d6 > d8 > d10) were labelled as up-up-up.

Table 1 shows an overview of the number of dynamic DEGs in induced and non-induced conditions in the female and male lines that were subjected to gonadal differentiation. Genes with a MaxPP value greater than 0.5 were considered to be high-confidence dynamic DEGs. In the induced condition of the female line about 8,193 genes were dynamic DEGs, of which 2,441 were high-confidence, whereas in the non-induced condition the figures were 7,127 and 2,595, respectively. In comparison, the induced condition of the male line showed 12,581 dynamic DEGs, of which 3,620 were high-confidence and in the non-induced condition there were 12,963 dynamic DEGs, of which 3,983 were high-confidence. The table also shows the overlap in dynamic DEGs between female and male lines in induced and non-induced time-series. We focused specifically on the DEGs induced in only female or male samples.

**Table 1:** Differential expression – time-series analysis comparison of the female and male lines

		Induced	Non-induced	
**Female**	**All dynamic DEGs (high confidence MaxPP > 0.5)**	8193 (2441)	7127 (2595)	
**Male**	**All dynamic DEGs (high confidence MaxPP > 0.5)**	12,581(3620)	12,963 (3983)	
		**Induced time-series**		**Non-induced time-series**
**Female-Male overlap**	**Only in female induced (high confidence)**	2004 (607)	**Only in female non-induced (high confidence)**	3808 (1513)
	**In females, induced or non-induced but not male induced (high confidence)**	4423 (1484)		
	**In females, induced AND non-induced (high confidence)**	2419 (877)		
	**Only in males (high confidence)**	2296 (834)	**Only in males non-induced (high confidence)**	9613 (2998)
	**In males, induced or non-induced but not female induced (high confidence)**	8779 (2682)		
	**In males, induced AND non-induced (high confidence)**	6483 (1848)		
	**Shared in females and males, induced, same pattern**	770 (294)	**Shared in females and males, non-induced, same pattern**	647 (313)
	**Shared in females and males, induced, different pattern**	2968 (738)	**Shared in females and males, non-induced, different pattern**	2641 (801)

About 2,004 genes were expressed in ‘Only in female induced’ samples, meaning that these genes showed dynamic changes in their expression pattern during gonadal differentiation of female cells upon *NR5A1* activation. Of these genes, 96 could be grouped in the up-up-up pathway and 40 of them showed significant upregulation (MaxPP > 0.5). These genes were mostly associated with ‘cortisol synthesis and secretion’ and ‘cAMP signalling’ KEGG pathways. Several of these genes, including *POMC*, *CCL22*, *NOV* (*CCN3*) and *ADCY4* have previously been associated with expression or action in the adrenal gland/adrenocortical cells [31–34]. The gene with the highest MaxPP in the female induced up-up-up pathway was *RP11-320G24.1*, followed closely by *POMC*. *RP11-320G24.1* is a long non-coding RNA (lncRNA) that is part of a tissue-specific co-expression gene regulatory network in the adrenal gland. It has been reported to have a positive correlation with *NR5A1*, amongst other genes [35]. Pro-opiomelanocortin (*POMC*), on the other hand, is precursor peptide of adrenocorticotrophic hormone (ACTH) [31].

**Fig. 4:**
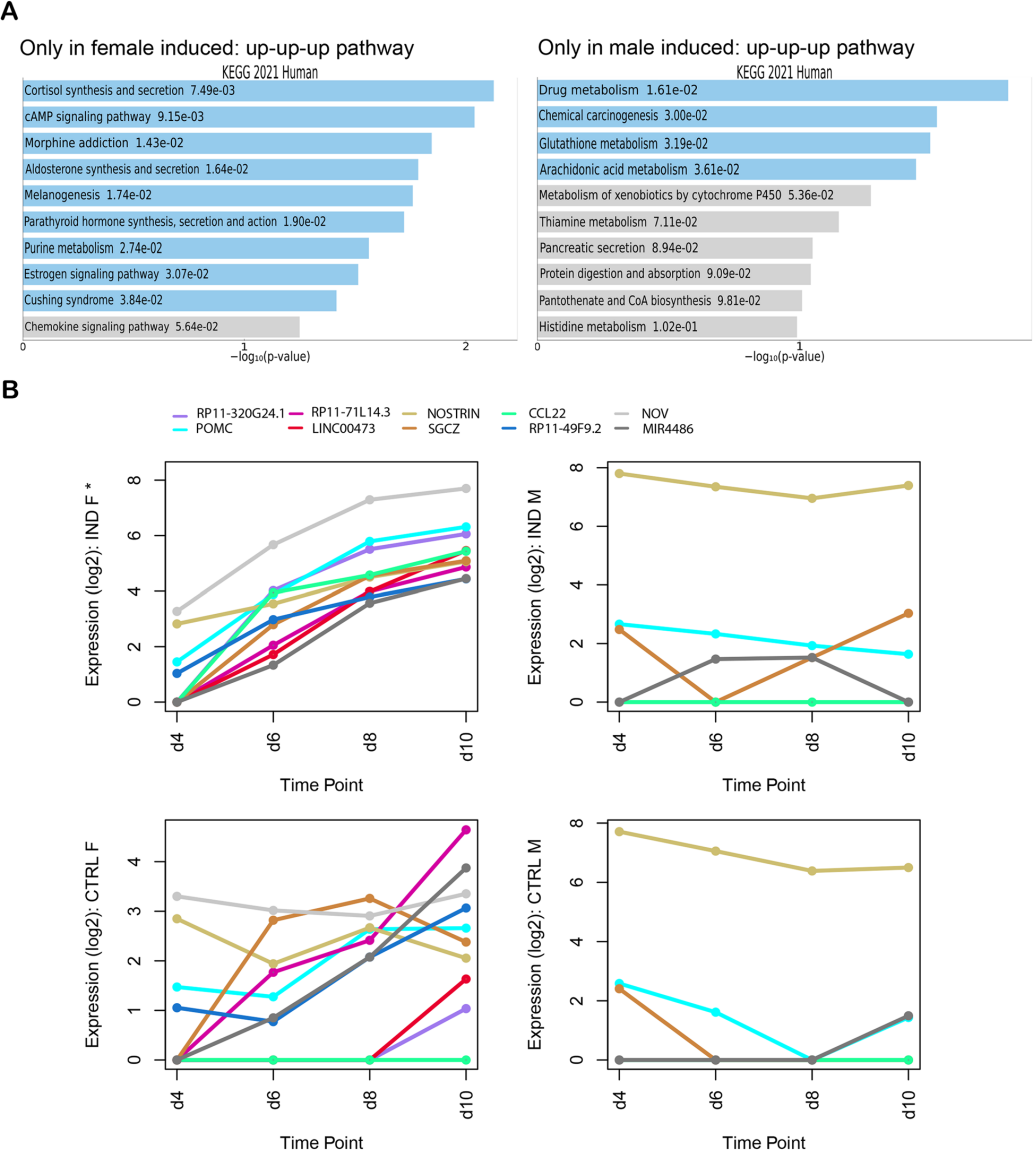
Time-series analysis demonstrated different dynamic changes in gene expression between female and male samples. **A** Bar charts showing the correlated KEGG 2021 human pathways of the continuously upregulated (up-up-up) genes in female induced samples across the different timepoints in gonadal differentiation. The bar charts show the top 10 enriched terms in the chosen sample set, along with their corresponding *p*-values. Coloured bars correspond to terms with significant *p*-values (< 0.05). **B** Log2 plots showing the top 10 continuously upregulated genes (*RP11-320G24.1*, *POMC*, *RP11-71L14.3*, *LINC00473*, *NOSTRIN*, *SGCZ*, *CCL22*, *RP11-491F9.2*, *NOV* and *MIR4486*) in the female induced samples (IND F) compared with their expression in control female (CTRL F), induced male (IND M) and control male (CTRL M) samples. The asterisk (*) symbolizes significant expression of these genes

In the ‘Only in male induced’ samples, 2,296 genes were expressed that showed dynamic changes in their expression patterns during male gonadal differentiation after *NR5A1* activation. A total of 133 genes could be grouped into the up-up-up pathway, 98 of which showed significant upregulation (MaxPP > 0.5). These genes were largely associated with ‘drug metabolism’ and ‘chemical carcinogenesis’ KEGG pathways. The gene with the highest MaxPP value in the up-up-up pathway was tescalcin (*TESC)*, a transcript expressed during the early stages of testicular differentiation in mice [36, 37] (Fig. 4A).

Figure 4B shows the expression patterns across the different timepoints of differentiation of the top 10 (10 highest MaxPP values) up-up-up genes from the ‘Only in female induced’ samples. The genes (*RP11-320G24.1*, *POMC*, *RP11-71L14.3*, *LINC00473*, *NOSTRIN*, *SGCZ*, *CCL22*, *RP11-491F9.2*, *NOV* and *MIR4486*) are plotted in log2 values and compared against their expression in female control, male induced and male control samples. As these genes belong to the up-up-up pathway of the ‘Only in female induced’ samples, they showed an upward trend in the female induced samples and a slight upward trend in the female control samples, but their expression remained constant and low in the male samples. Moreover, some of the genes were not even expressed in the male samples (Fig. 4B).

Taken together, different genes showed continuous upregulation in the female induced and male induced samples.

## Discussion

In this study, we showed that inducing *NR5A1* in a female (46,XX) hESC line, which has been differentiated to bipotential gonadal-like cells, steered these cells towards a steroidogenic-like cell population, but did not seem to promote ovarian differentiation. Bipotential gonadal markers were upregulated by day 8 of differentiation, which is a result of the differentiation process rather than the effect of *NR5A1* induction. By day 10 of gonadal differentiation, we saw upregulation of early ovarian markers such as *RSPO1*, *WNT4* and *INHA*, but for *RPSO1* and *WNT4* this upregulation was not due to *NR5A1* induction but again a result of the differentiation protocol itself. *NR5A1* induction, however, significantly upregulated the expression of the steroidogenic genes *STAR*, *CYP11A1, CYP17A1, HSD3B2 and HSD17B1*. Interestingly, expression of the aromatase gene (*CYP19A1*), which is expressed by the granulosa cells [38], was relatively low and was decreased significantly on day 6 of differentiation due to *NR5A1* induction and continued to decrease over the course of differentiation. These are the first indicators that *NR5A1* directs the bipotential gonadal-like cells towards a steroidogenic-like cell population in a female cell line.

These interpretations of the results are also supported by our bulk RNA-seq data. According to the KEGG pathway analyses, *NR5A1*-induced pathways seem to be predominantly associated with ‘cortisol and aldosterone synthesis and secretion’ and not with ovarian-type steroidogenic pathways. Moreover, the top 20 DEGs in the non-induced versus the induced female samples at day 6 of differentiation were *HPGD*, *NR5A1*, *MYH6*, *CPN2*, *SIGLEC11*, *SULT2A1*, *PTPN7*, *GNRHR*, *KRT81*, *KRT86*, *CASR*, *WNT6*, *GSTA1*, *GSTA2*, *KCNT1*, *MIR202HG*, *SCARA5*, *FFAR1*, *CLEC4E* and *AICDA*. Among these, HPGD, *SULT2A1*, *GNRHR* and *GSTA1* have previously been associated with adrenal steroidogenesis [29, 39]. Time-series analysis further confirmed the presence of a steroidogenic cell-like population in the female induced samples, as continuously upregulated genes were again mostly associated with ‘cortisol synthesis and secretion’. Together, these results suggest that *NR5A1* induction in a female stem-cell line promotes the generation of a predominantly steroidogenic-like cell population possibly resembling an adrenal cell type instead of a more mature female gonadal-like cell type, as is the case in gonadal differentiation of the male cell line [26].

RNA-seq further highlighted some key differences observed between gonadal differentiation of the female and male lines. The PCAs showed that PC2 in gonadal differentiation of the female line explains the greater variation in the data than PC2 in gonadal differentiation of the male line [26] (PC2 values 34% and 18%, respectively). As both PCs depicted similar levels of total variation in the respective data sets, these data suggest that *NR5A1* induction has a wider impact on the female line. Pairwise DE analysis showed that in the female cell line, the highest number of DEGs could be found at day 6 of gonadal differentiation, while in the male cell line this was on day 10. Thus, the largest effect due to *NR5A1* induction seems to occur earlier during gonadal differentiation of the female line than of the male line. The pairwise DE analysis also showed that there were in general more upregulated DEGs during gonadal differentiation of the female line, whilst downregulated DEGs dominated in the male. Next, DE time-series analysis was performed and compared with the corresponding male DE time-series analysis. Interestingly, different genes showed dynamic changes in their expression patterns in female versus male induced samples. This further demonstrates the divergence in differentiation patterns in female and male hPSCs. Whereas in female induced samples *RP11-320G24 and POMC* were the two most highly continuously upregulated genes, *TESC* was the top continuously upregulated gene in the male induced samples. As mentioned previously, *RP11-320G24.1* is part of an adrenal gland tissue-specific co-expression gene regulatory network and has been shown to have a positive correlation with *NR5A1* [35]. *POMC* is generally found in the hypothalamus, yet mRNA transcripts have also been found in the normal adrenal gland and the ovary [40]. *POMC* can be cleaved and produces *ACTH*, which regulates adrenal steroidogenesis. While *ACTH* production mainly takes place within the corticotrophic cells of the anterior pituitary, its expression has also been found in the adrenal medulla, the brain, the skin and the placenta [31]. *TESC*, on the other hand, is a known testicular transcript expressed during the early stages of testicular differentiation [36, 37].

As for potential targets of *NR5A1*, gonadotrophin releasing hormone receptor (*GNRHR)* might be a putative target for the processes of gonadal differentiation of the female line and steroidogenesis. *GNRHR* has been known to be expressed in normal human reproductive tissues such as the ovary [41] and additionally its mRNA was highly expressed in our *NR5A1* induced cells. Moreover, gonadotrophin releasing hormone (*GNRH*) has been known to directly regulate the activity of steroidogenic enzymes such as *CYP19A1* [42]. Thus, it is possible that *GNRH* can mediate the steroidogenic effect of *NR5A1*. In the male hPSC model, scavenger receptor class a member 5 (*SCARA5*) had already been recognised as a putative target of *NR5A1* by Sepponen et al. [26]. Our data supports the finding of *SCARA5* being a putative target of *NR5A1*, as *SCARA5* is among the top 20 DEGs in the non-induced and induced genes at day 6 of differentiation of the female line.

As the focus of our study was to investigate the impact of *NR5A1* expression on female bipotential gonadal-like cells, the identity of these steroidogenic-like cells was not thoroughly elucidated. The low *CYP19A1* mRNA and protein expression, together with the general increase in steroidogenic gene expression, might be an indication of the progression of bipotential gonadal-like cells towards an adrenocortical population, as expression of *CYP19A1* has been reported to be relatively low in the normal adult adrenal gland [43]. However, our cells represent early fetal cells and not adult cells, and therefore certain genes (such as *CYP19A1*) could be expressed at levels different to those reported in adults. Moreover, a significant upregulation of *INHA* following *NR5A1* induction was observed. *INHA* is known to be produced by human granulosa cells, but also by the steroidogenic theca cells [44, 45]. Furthermore, ‘ovarian steroidogenesis’ is one of the KEGG pathways that was significantly expressed during DE analysis on days 6, 8 and 10 of differentiation. Therefore, the presence of some female steroidogenic cells, theca cells, in the heterogenous pool of cells, cannot be excluded. Additionally, in male gonadal differentiation the male pre-gonadal cells also strongly express steroidogenic enzymes and secrete steroid hormones [26]. Thus, it might be possible that the steroidogenic cells observed here could represent (at least partly) pre-granulosa cells in the early stages of development. Most likely, the steroidogenic-like cell population might be a mixture of adrenocortical and thecal cells amongst other cell types.

Our results are in line with those reported by Smith et al. [46], where they created a *CYP17A1* conditional knock-out mouse (cKO) model, where *NR5A1* was depleted in the steroidogenic Leydig cells and theca cells of mature mice. With this *CYP17A1* cKO model they showed that *NR5A1* depletion in Leydig cells of mature male mice can result in male infertility, whilst depletion of *NR5A1* in theca cells of mature female mice mainly caused impaired steroidogenesis, with fertility being preserved. Moreover, in 2019 Stévant et al. [47] showed that *NR5A1*-positive progenitors in fetal female (XX) mice give rise to both pre-granulosa cells and steroidogenic precursor cells. This suggests that indeed *NR5A1* might not be as essential in female gonadal development as it is for male gonadal development but has a more prominent role in steroidogenesis.

Limitations of this study include the unclear identity of the cell type obtained by the end of our gonadal differentiation protocol. Although it is fairly certain that the generated cells are steroidogenic, it remains uncertain what type of steroidogenic cells they actually are (ovarian, testicular or adrenal). Moreover, it must be noted that there was a lack in upregulation of specific ovarian markers during gonadal differentiation. Thus, despite generating bipotential gonadal-like and steroidogenic cells, our protocol (including *NR5A1* activation) did not seem to completely reflect the developmental process of female gonadal cells. Additionally, it is very clear that the cell population was relatively heterogeneous even though the cells were directed towards a specific cell lineage. As a technical factor, the expression levels of *NR5A1* differed in the female and male lines, with levels in the female line being 5–10 times higher, which may have contributed to the gene expression observed. Another limitation of this study, and related to expression levels, is the use of artificial induction of *NR5A1*. As yet, we have not been able to establish a differentiation protocol where *NR5A1* could be induced endogenously without the use of genome-editing techniques. Thus, we do not know how high the natural gene expression level of *NR5A1* during gonadal differentiation might be. This makes it difficult to compare the in vitro with the in vivo situation and thus our current protocol remains unsuitable for clinical applications where patient lines could be reprogrammed and differentiated.

## Conclusions

We conclude that during gonadal differentiation of a female hPSC line, *NR5A1* seems to steer hESC-derived bipotential gonadal-like cells towards a heterogeneous, steroidogenic-like cell population. This differs from gonadal differentiation of a male hPSC line, where *NR5A1* directs the hiPSCs towards a more mature, gonadal-like cell type. Thus, *NR5A1* is a necessary factor in gonadal differentiation of a female cell line yet does not seem to be sufficient to direct the gonadal-like cells towards a granulosa cell-like state. The findings in this study could aid in elucidation of the mechanisms underlying human fetal gonadal development.

## Materials and methods

### Cell culture

All cells were cultured in a humidified incubator supplied with 5% CO_2_ at 37 °C. H9-*NR5A1*-DDdCas9VP192 (clone 10) hESCs were cultured on Geltrex® LDEV-free human embryonic stem cell-qualified-reduced growth factor basement membrane matrix (Gibco, Thermo Fisher Scientific, Waltham, MA, USA) or growth factor-reduced Matrigel (Corning, NY, USA) in tissue culture-treated dishes (Corning, NY, USA) in Essential 8 (E8) medium (Thermo Fisher Scientific, Waltham, MA, USA). The cells were routinely passaged every 4–5 days. For routine passaging, the cells were incubated in 0.5 mM EDTA (Invitrogen™, Thermo Fisher Scientific, CA, USA) in phosphate-buffered saline (PBS) for 4 min.

Differentiation of the hESCs was performed as previously described (condition M, Sepponen et al., 2017). Undifferentiated hESCs were dissociated with 0.5 mM EDTA, counted, and seeded at a density of 2.24 × 10^5^ cells/cm^2^ on tissue culture-treated 12-well plates (Corning, NY, USA) for the collection of RNA, or on *µ*-slide 4 wells (IBIDI, Gräfelfing, Germany) for immunofluorescence staining. Both 12-well culture plates and *µ*-slides were coated with human placental-derived type I collagen (Corning, NY, USA). Prior to seeding the cells were centrifuged at 60 × g for 5 min and resuspended in intermediate mesoderm (IM) medium [DMEM/F12 (Gibco™, Thermo Fisher Scientific, Waltham, MA, USA) + Glutamax (Thermo Fisher Scientific, Waltham, MA, USA) supplemented (2%) with B-27™ Supplement (Thermo Fisher Scientific, Waltham, MA, USA)]. The medium was additionally supplemented with 10 µM rho kinase inhibitor (ROCKi, Y-27,632 2HCL, Selleckchem, Houston, TX, USA), activin A (Q-kine, Cambridge, UK) at 100 ng/mL, 5 µM GSK-3α/β inhibitor (CHIR-99,021, Selleckchem, Houston, TX, USA) and 2 µM BMP inhibitor (dorsomorphin, Selleckchem, Houston, TX, USA). On the following day, the media were changed to IM medium supplemented with BMP7 (Peprotech, Cranbury, NJ, USA) at 10 ng/mL and 3 µM CHIR-99,021. After 24 h, the medium was replaced with IM medium containing 2 µM dorsomorphin and 3 µM CHIR-99,021, which was kept on the cells for 48 h. Between media changes, the cells were washed once with PBS containing Mg^2+^ and Ca^2+^ (Gibco™, Thermo Fisher Scientific, Waltham, MA, USA). On day 4 until day 10 of differentiation, *NR5A1* was induced by supplementing IM medium with DOX (Sigma-Aldrich, Saint Louis, MO, USA) at 1 µg/mL and 1 µM TMP (Sigma-Aldrich, Saint Louis, MO, USA). Most of the induction medium was changed daily.

### Generating H9-*NR5A1*-DDdCas9Vp192 hESCs

The H9 cell line (WiCell Research Institute, Madison, WI, USA) was engineered to express a DDdCas9-VP192 construct under the control of a DOX-inducible promoter.

PiggyBac plasmids PB-GG-*NR5A1*-PGK-Puro (containing four guides targeting *NR5A1* promoter, described by Sepponen et al., 2022), PB-tight-DDdCas9VP192-T2A-GFP-IRES-Neo (RRID:Addgene_ 102,889, Weltner et al., 2018) and PB-CAG-rtTA (kindly provided by the Biomedicum Stem Cell Centre, University of Helsinki, Helsinki, Finland), together with a PB transposase (kindly provided by the Biomedicum Stem Cell Centre, University of Helsinki, Helsinki, Finland), were simultaneously introduced to H9 hESCs. Cells were electroporated as described previously (Sepponen et al., 2022). Briefly, H9 cells were incubated with 10 µM Y-27,632 2HCl in E8 Basal Medium for 4 h prior to dissociation with StemPro® Accutase® (GibcoTM, Thermo Fisher Scientific, Waltham, MA, USA). They were rinsed with PBS and trypsinised with Accutase for 5 min at 37 ˚C. Subsequently, dissociated cells were resuspended in 5% fetal bovine serum (FBS) in PBS and counted. The cells were pelleted and resuspended in R buffer (Invitrogen™, Thermo Fisher Scientific, CA, USA). Two million cells, 1 µg PB-GG-NR5A1-PGK-Puro, 1 µg PB-tight-DDdCas9VP192-T2A-GFP-IRES-Neo, 0.5 µg PB-CAG-rtTA and 0.5 µg PB transposase were used for each transfection, using the NeonTM Transfection System (Invitrogen™, Thermo Fisher Scientific, CA, USA). The electroporation program settings have been described previously (Balboa et al., 2015 [48]; Sepponen et al., 2022 [26]). The cells were plated on Geltrex® (Gibco, Thermo Fisher Scientific, Waltham, MA, USA)-coated dishes for 24 h, followed by a change of medium (E8).

Selection of colonies with guides targeting *NR5A1* was started 72 h after transfection with puromycin at 0.5 µg/mL for 2 days and at 0.25 µg/mL for 4 days. Cells were cultured in fresh E8 medium without antibiotics for 4 days and passaged. Following this, colonies containing DDdCas9 were selected with G418 sulphate (Merck Millipore, Darmstadt, Germany) at 0.25 mg/mL and DOX at 0.5 µg/mL for 3 days, followed by selection with G418 at 0.1 mg/mL and DOX at 0.5 µg/mL for an additional 3 days. To obtain colonies from single clones, cells were sorted with Influx equipment (BD, Biosciences) into microwell 96-well plates (Nunc, Thermo Fisher Scientific, Rochester, NY, USA), picked and expanded as described previously (Sepponen et al., 2022 [26]).

### Immunofluorescence and confocal microscopy

At day 10 of differentiation, cells on *µ*-slide 4 wells were washed once with PBS + Mg^2+^, +Ca^2+^ and fixed with 4% paraformaldehyde at room temperature for 15 min, after which they were washed three times with PBS (Medicago, Uppsala, Sweden). Next, the cells were permeabilised with 0.5% Triton® X-100 (Fisher Scientific, Waltham, MA, USA), washed three times with 0.1%Tween® (Fisher Scientific, Waltham, MA, USA) in PBS and blocked with UltraVision Protein Block (Fisher Scientific, Waltham, MA, USA) at room temperature for 10 min. The cells were then incubated with primary antibodies diluted in 0.1% Tween in PBS overnight at 4 °C: mouse monoclonal anti-SF1 (R and D Systems, Cat# PP-N1665-00, RRID:AB_2251509, 1:250), goat polyclonal anti-FOXL2 (Novus Biologicals, Cat# NB100-1277, RRID:AB_2106187, 1:250), goat polyclonal anti-GATA4 (Santa Cruz Biotechnology, Cat# sc-1237, RRID:AB_2108747, 1:250), mouse monoclonal anti-WNT4 (Santa Cruz Biotechnology, Cat# sc-376,279, RRID:AB_10986273, 1:250), mouse monoclonal anti-CYP19A1 (Santa Cruz Biotechnology, Cat# sc-374,176, RRID:AB_10986411, 1:500), and mouse monoclonal anti-STAR (Santa Cruz Biotechnology, Cat# sc-166,821, RRID:AB_2197661, 1:250). Subsequently, the cells were washed three times with 0.1% Tween in PBS. Next, cells were incubated with the following secondary antibodies diluted 1:1000 in 0.1% Tween in PBS: Alexa Fluor 488 donkey anti-mouse immunoglobulin G (IgG) (Molecular Probes, Cat# A-21,202, RRID:AB_141607) or Alexa Fluor® 594 donkey anti-goat IgG (Thermo Fisher Scientific, Cat# A-11,058, RRID:AB_2534105). Incubation was carried out for 45 min at room temperature in the dark. Next, the cells were washed twice with 0.1% Tween in PBS. Nuclei were labelled with 4’,6-diamidino-2-phenylindole (DAPI) dilactate (Invitrogen, Thermo Fisher Scientific, Cat#D3571, RRID:AB_2307445) at a 1:1000 ratio in 0.1% Tween in PBS for 10 min in the dark and washed twice with 0.1% Tween in PBS.

Images were captured with a TCS SP8 confocal microscope with a white laser (Leica Microsystems, Mannheim, Germany) using an 812 × 812 format and an HC PL APO CS2 40x/1.30 oil objective. Images were processed using Fiji version 2.3.0 equipment (http://fiji.sc).

### Real time quantitative polymerase chain reactions (RT-qPCRs)

Total RNA was isolated using Nucleospin® RNA kits (Macherey-Nagel, Düren, Germany). Genomic DNA was removed separately by using ribonuclease-free deoxyribonuclease (Promega, Madison, WI, USA), after which RNA was again purified (Nucleospin® RNA clean-up kits). Reverse transcription into complementary DNA was carried out with the use of Moloney murine leukaemia virus reverse transcriptase (Promega. Madison, WI, USA), oligo(dt)18 primers, random hexamer primers, RiboLock RNAse Inhibitor, and a mixture of four deoxynucleotide triphosphates (all from Thermo Fisher Scientific, Waltham, MA, USA). cDNA was combined with a mixture of 5× HOT FIREPol® EvaGreen® qPCR Mix Plus (no ROX) (Solis Biodyne, Tartu, Estonia) and a primer mix containing forward and reverse primers (Metabion, Planegg, Germany) (primer sequences are listed in Table 2). Relative messenger RNA (mRNA) expression levels were analysed with Lightcycler® 96 equipment (Roche Diagnostics, Basel, Switzerland). For relative quantification of gene expression, the ΔΔCt method (Livak and Schmittgen, 2001) was employed. Expression levels were normalized to those of the housekeeping gene peptidylprolyl isomerase G (*PPIG*) and presented relative to expression in undifferentiated H9 cells.

**Table 2:** RT-qPCR primer pair sequences

Primer Name	Sequence of 5′ Primer (Forward)	Sequence of 3′ Primer (Reverse)
NR5A1	CAGGAGTTTGTCTGCCTCAA	GCACAGGGTGTAGTCAAGCA
GATA4	CAGGCGTTGCACAGATAGTG	CCCGACACCCCAATCTC
WT1	GGCAGCACAGTGTGTGAACT	CCAGGCACACCTGGTAGTTT
FOXL2	TTTGTCCCCTCAGTTTATGTCC	TGAATTTGGGCAGGAGACG
WNT4	GATGTGCGGGAGAGAAGCAA	ATTCCACCCGCATGTGTGT
RSPO1	GCAACCCCGACATGAACAAG	CAAGCCCTCCTTACACTTGG
STAR	GAGTCAGCAGGACAATGGGG	CGCTCCACGAGCTCTTCATA
CYP11A1	TGGTGACAATGGCTGGCTAAA	ATAAACCGACTCCACGTTGC
CYP17A1	TCAGCCGCACACCAACTATC	GCAAACTCACCGATGCTGGA
CYP19A1	GTGGCCCATGGCATTTTATA	GAGCTCTACTGGGGAACCAG
HPGD	CAGCCGGTTTATTGTGCTTC	CAGTCTCACACCACTGTTCATA
HSD3B2	GAGGCAGTAAGGACTTGGACT	TGACCCAGAAGAGGGCGTAA
HSD17B1	TGATGGGGCTGCCTTTCAAT	ATCAGGCTCAAGCTGTGGAC
GSTA1	GATGTTCCAGCAAGTGCCAAT	CAGGTGGACATACGGGCA
GSTA2	ATCCTTCTTCTGCCCTTTAGTC	GTCTTGTCCGTGGCTCTTTA
WNT6	AACAGGACATTCGGGAGACG	CAGCTCGCCCATAGAACAGG
B4GALNT2	TACCAAGACTTTCCTCCGCC	AGCAAACCAACCCTTCCCAA
GNRHR	AGTTTTTCACAATGGTGGCATCAA	TGGCAAATGCAACCGTCATT
PPIG	TCTTGTCAATGGCCAACAGAG	GCCCATCTAAATGAGGAGTTG

### RNA sequencing (RNA-seq)

Cells cultured in triplicate in 12-well culture plates were lysed with QlAzol Lysis Reagent (Qiagen, Hilden, Germany) at days 4, 6, 8 and 10 of differentiation. Total RNA was isolated using miRNeasy Mini Kits (Qiagen, Hilden, Germany). Additional on-column DNase Digestion with the RNase-Free DNase Set (Qiagen, Hilden, Germany) was performed. Determination of RNA quality, library preparation and sequencing were performed by the Sequencing unit of the Institute for Molecular Medicine Finland FIMM Technology Centre, University of Helsinki, Finland. Total RNA quality checks were carried out by using LabChip GX Touch RNA assays (PerkinElmer, USA) for RNA integrity checks and Qubit BR RNA assays (Thermo Fisher Scientific, USA) for total RNA quantification. Library preparation from 800 ng of total RNA was performed according to Illumina methodology (stranded total RNA prep with Ribo-Zero Plus Reference Guide, Illumina, San Diego, CA, USA). Library quality checks were performed using LabChip GX Touch HT High Sensitivity assays (PerkinElmer, USA). The libraries were quantified for sequencing using KAPA Library Quantification Kits (KAPA Biosystems, Wilmington, MA, USA). Sequencing was performed by using Illumina equipment (NovaSeq system using an S1 flow cell with lane divider, Illumina, San Diego, CA, USA). Read length for the paired-end run was 2 × 151 bp.

Data pre-processing was performed at FIMM. Quality control of raw sequence data was performed with FastQC v0.11.9. Trim-Galore v0.6.5 was used for adapter trimming and quality filtering. RNA-seq reads were then aligned against the human reference genome (GRCh38 release 82) using STAR-2.7.6a. Then, the quality of RNA-Seq quality matrices was inspected with RNA-SeQC v2.4.2 and the quality of RNA-Seq data was assessed with RSeQC v4.0.0. FeatureCounts software was then used to summarise the read distribution over several genomic features.

### Data analysis

The gene-by-sample expression data matrix, prepared by merging the output of FeatureCounts, was used for pairwise DE analyses with DESeq2 (v1.24, Love et al., 2014), to determine DEGs at each timepoint, and for time-series DE analysis with EBSeq-HMM (v1.18, Leng et al., 2015), to determine the expression dynamics of the DEGs throughout the time-course. Default settings for data normalization were used for both DESeq2 and EBSeqHMM.

Pairwise comparisons were conducted by comparing the induced and non-induced samples at days 6, 8, and 10. Additionally, samples in the same conditions were compared between timepoints. DEGs with adjusted *P* < 0.05 were then used in downstream pathway analyses. Time-series DE analyses were conducted separately for the *NR5A1*-induced and non-induced samples separately with the timepoints used as the ordering condition in EBSeqHMM. There are three possible transitions across the four ordered timepoints (days 4, 6, 8, 10) in our data, at which genes can attain one of three states: upregulation (U), downregulation (D) or stable expression (S or EE), making up 27 possible paths genes can follow in this time course (U-U-U, D-D-D, etc.). EBSeqHMM estimates the posterior probabilities (PPs) for all the possible paths and identifies genes with a non-constant expression profile over the time-course. Genes with significant DE in at least one transition between timepoints and having a PP of < 0.05 of remaining constant are identified as dynamically DEGs. The path with the highest PP (MaxPP) is assigned to each DEG. To compare the dynamically DEGs with the results from the male gonadal differentiation reported by Sepponen et al. [26] we re-analysed the male gonadal RNA-seq data with EBSeqHMM and evaluated the overlap of the dynamically DEGs between the female and male gonadal cell lines. Dynamically DEGs in our female *NR5A1*-induced data that were not detected as dynamically DEGs in our female non-induced data as well as the male *NR5A1*-induced data were identified as ‘Only in Female Induced’ DEGs (genes that show statistically significant dynamic DE profiles only in *NR5A1*-induced female cell line samples). Similarly, genes with statistically significant dynamic DE patterns in the *NR5A1*-induced male cell line samples (and not in the induced female cell line nor the non-induced male cell line samples) were identified as ‘Only in Male Induced’ DE genes. Additionally, we also identified dynamically DEGs that were detected in both cell lines (with either the same dynamic pattern or different), and those that were sex-specific having dynamically DE profiles only in one sex regardless of *NR5A1* induction status.

### GO enrichment analysis

GO enrichment analyses were performed using the online tool ENRICHR from the Maayan lab (ENRICHR, RRID:SCR_001575, https://maayanlab.cloud/Enrichr/).

### Statistical analysis

Statistical analysis was performed with both GraphPad Prism version 9.2.0 (San Diego, CA, USA) and IBM SPSS 29 Statistics software (Chicago, IL, USA). Normal distribution of the data was assessed by using the Shapiro–Wilk normality test. Two-way ANOVA was used to assess the significance between the non-induced and induced samples across the different timepoints during differentiation. In correction for multiple comparison, Sidak’s post-hoc test was used. P values less than 0.05 were considered statistically significant. Data are shown as mean ± SEM.

## Supplementary Information

**Additional file 1: Supplementary figure 1. **Bulk RNA-sequencing analyses showed differences between the control (-DOX-TMP) and induced (+DOX+TMP) conditions. A. Principal component analysis (PCA) comparing samples from different days of differentiation and control (CTRL, -DOX –TMP) and induced (IND, +DOX +TMP) conditions. d; day. B. Flowchart comparing the number of differentially expressed (black), coding (orange), non-coding (blue), upregulated (green) and downregulated (red) genes in induced (+DOX+TMP) and non-induced (-DOX-TMP) conditions at different timepoints in both female and male gonadal differentiation. d; day. **Supplementary figure 2. **Differentially expressed upregulated genes were mostly associated with (adrenal) steroidogenesis. A. RT-qPCR analysis showed that *NR5A1 *induction upregulated the markers *HPGD, GSTA1*, *GSTA2*, *WNT6, B4GALNT2* and *GNRHR*. Data are reported as mean ± SEM, n=3 biological replicates. Two-way ANOVA; 0.12 (ns), 0.033 (*), 0.002 (**), < 0.001 (***). d; day. B. Bar charts showing the correlated KEGG 2021 human pathways of the female DE genes between control (CTRL, -DOX -TMP) and induced (IND, +DOX +TMP) conditions at days 6, 8 and 10 of gonadal differentiation. The bar charts show the top 10 enriched terms in the chosen library, along with their corresponding p-values. Coloured bars correspond to terms with significant p-values (< 0.05). An asterisk (*) next to a p-value indicates the term also had a significant adjusted *p*-value (< 0.05). **Supplementary figure 3. **Adrenal, testicular, and ovarian steroidogenesis markers were upregulated upon *NR5A1* induction. Comparison of the expression of RNA-sequenced non-induced (CTRL, -DOX-TMP) and induced (IND, +DOX+TMP) samples. Known adrenal, testicular and ovarian steroidogenic markers were upregulated upon *NR5A1* induction. Each square represents the expression of a specific gene within a technical replicate. The intensity of gene expression is indicated by a colour scale based on row z-scores (red, highest expression levels; blue, lowest expression levels). d, day.

## Data Availability

The datasets generated and/or analysed during the current study are available in the NCBI Gene Expression Omnibus (GEO) repository and can be accessed through GEO Series accession number GSE236653. All material unique to this study is available from the corresponding author on reasonable request.

## Abbreviations

B4GALNT2: Beta-1,4-N-acetyl-galactosaminyltransferase 2
CYP11A1: Cytochrome p450 family 11 subfamily A member 1
CYP17A1: Cytochrome p450 family 17 subfamily A member 1
CYP19A1: Cytochrome p450 family 19 subfamily A member 1
DAPI: 4',6-diamidino-2-phenylindole
DE: Differential expression/differentially expressed
DEG: Differentially expressed gene
DOX: Doxycycline hyclate
DSD: Differences in sex development
EMX2: Empty spiracles homeobox 2
FBS: Fetal bovine serum
FOXL2: Forkhead box protein L2
GATA4: Gata binding protein 4
GSTA1: Glutathione S-transferase A1
hESC: Human embryonic stem cell
hiPSC: Human induced pluripotent stem cell
HPGD: 15-hydroxyprostaglandin dehydrogenase
HSD3B2: Hydroxy-delta-5-steroid dehydrogenase
HSD17B1: Hydroxysteroid 17-beta dehydrogenase 1
hPSC: Human pluripotent stem cell
IM: Intermediate mesoderm
INHA: Inhibin subunit α
KO: Knock-out
LHX9: Lim homeobox 9
NEAA: Non-essential amino acid
NR5A1: Nuclear receptor subfamily 5 group A member 1
PBS: Phosphate-buffered saline
PCA: Principal component analysis
POMC: Pro-opiomelanocortin
PPIG: Peptidylprolyl isomerase G
PS: Primitive streak
RT-qPCR: Real time quantitative polymerase chain reaction
ROCKi: Rho-kinase inhibitor
RSPO1: R-spondin 1
SF-1: Steroidogenic factor 1
SRY: Sex-determining region on the Y chromosome
STAR: Steroidogenic acute regulatory protein
TMP: Trimethoprim
WNT4: Wnt family member 4
WNT6: Wnt family member 6
WT1: Wilms’ tumour suppressor 1
