# Correction: CRISPR/Cas9-mediated activation of *NR5A1* steers female human embryonic stem cell-derived bipotential gonadal-like cells towards a steroidogenic cell fate

**DOI:** 10.1186/s13048-025-01655-w

**Published:** 2025-04-16

**Authors:** Laura Danti, Karolina Lundin, Kirsi Sepponen, Dawit A. Yohannes, Juha Kere, Timo Tuuri, Juha S. Tapanainen

**Affiliations:** 1 Department of Obstetrics and Gynecology, University of Helsinki and Helsinki University Hospital, Helsinki, 00290 Finland; 2 Research Programs Unit, Translational Immunology & Department of Medical and Clinical Genetics, University of Helsinki, Helsinki, 00290 Finland; 3 Folkhalsan Research Centre, Stem Cells and Metabolism Research Program, University of Helsinki, Helsinki, 00290 Finland; 4 Department of Biosciences and Nutrition, Karolinska Institutet, Huddinge, 14183 Sweden; 5 Department of Obstetrics and Gynecology, HFR– Cantonal Hospital of Fribourg and University of Fribourg, Fribourg, 1708 Switzerland

**Correction: J Ovarian Res 16, 194 (2023)**

10.1186/s13048-023-01264-5

Following publication of the original article [1], the author reported, that the second affiliation of Laura Danti was not correctly associated to her name in the published article.

**Incorrect**:

Laura Danti^1^

^1^ Department of Obstetrics and Gynecology, University of Helsinki and Helsinki University Hospital, Helsinki 00290, Finland

**Correct**:

Laura Danti^1,4^

^1^ Department of Obstetrics and Gynecology, University of Helsinki and Helsinki University Hospital, Helsinki 00290, Finland

^4^ Department of Biosciences and Nutrition, Karolinska Institutet, Huddinge 14,183, Sweden

The original article [1] has been corrected.
