# Supplementary material for: CRISPR/Cas9-mediated activation of NR5A1 steers female human embryonic stem cell-derived bipotential gonadal-like cells towards a steroidogenic cell fate
Source: J Ovarian Res. 2023 Sep 20;16:194. doi: 10.1186/s13048-023-01264-5 (PMC10510196; doi:10.1186/s13048-023-01264-5)

**Supplementary figure 1: Bulk RNA-sequencing analyses showed differences between the control (-DOX-TMP) and induced (+DOX+TMP) conditions.**

**A.** Principal component analysis (PCA) comparing samples from different days of differentiation and control (CTRL, -DOX –TMP) and induced (IND, +DOX +TMP) conditions. d; day. **B.** Flowchart comparing the number of differentially expressed (black), coding (orange), non-coding (blue), upregulated (green) and downregulated (red) genes in induced (+DOX+TMP) and non-induced (-DOX-TMP) conditions at different timepoints in both female and male gonadal differentiation. d; day.


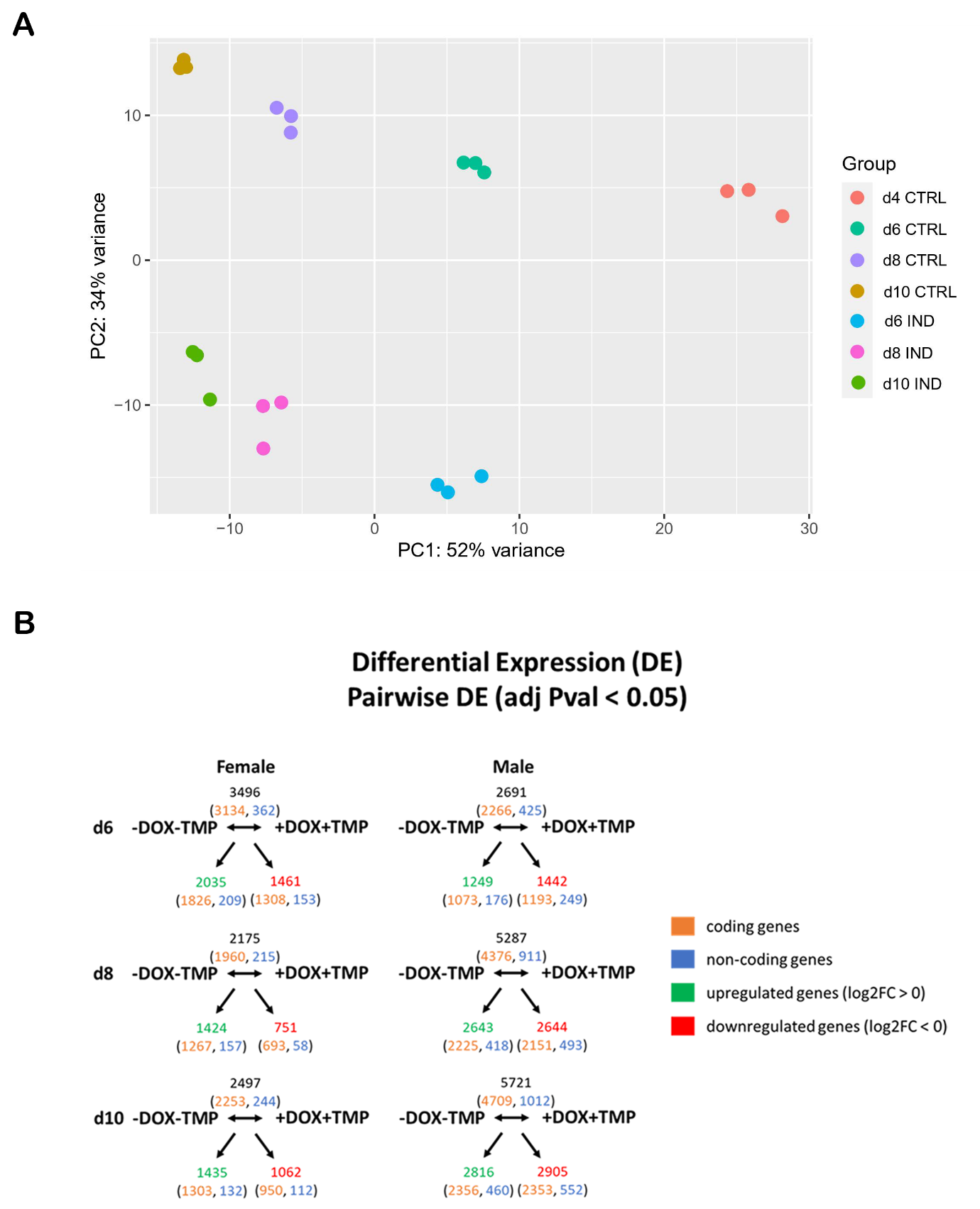


**Supplementary figure 2: Differentially expressed upregulated genes were mostly associated with (adrenal) steroidogenesis.**

**A.** RT-qPCR analysis showed that *NR5A1* induction upregulated the markers *HPGD, GSTA1*, *GSTA2*, *WNT6, B4GALNT2* and *GNRHR*. Data are reported as mean ± SEM, n=3 biological replicates. Two-way ANOVA; 0.12 (ns), 0.033 (*), 0.002 (**), < 0.001 (***). d; day. **B.** Bar charts showing the correlated KEGG 2021 human pathways of the female DE genes between control (CTRL, -DOX -TMP) and induced (IND, +DOX +TMP) conditions at days 6, 8 and 10 of gonadal differentiation. The bar charts show the top 10 enriched terms in the chosen library, along with their corresponding p-values. Coloured bars correspond to terms with significant p-values (< 0.05). An asterisk (*) next to a p-value indicates the term also had a significant adjusted p-value (< 0.05).


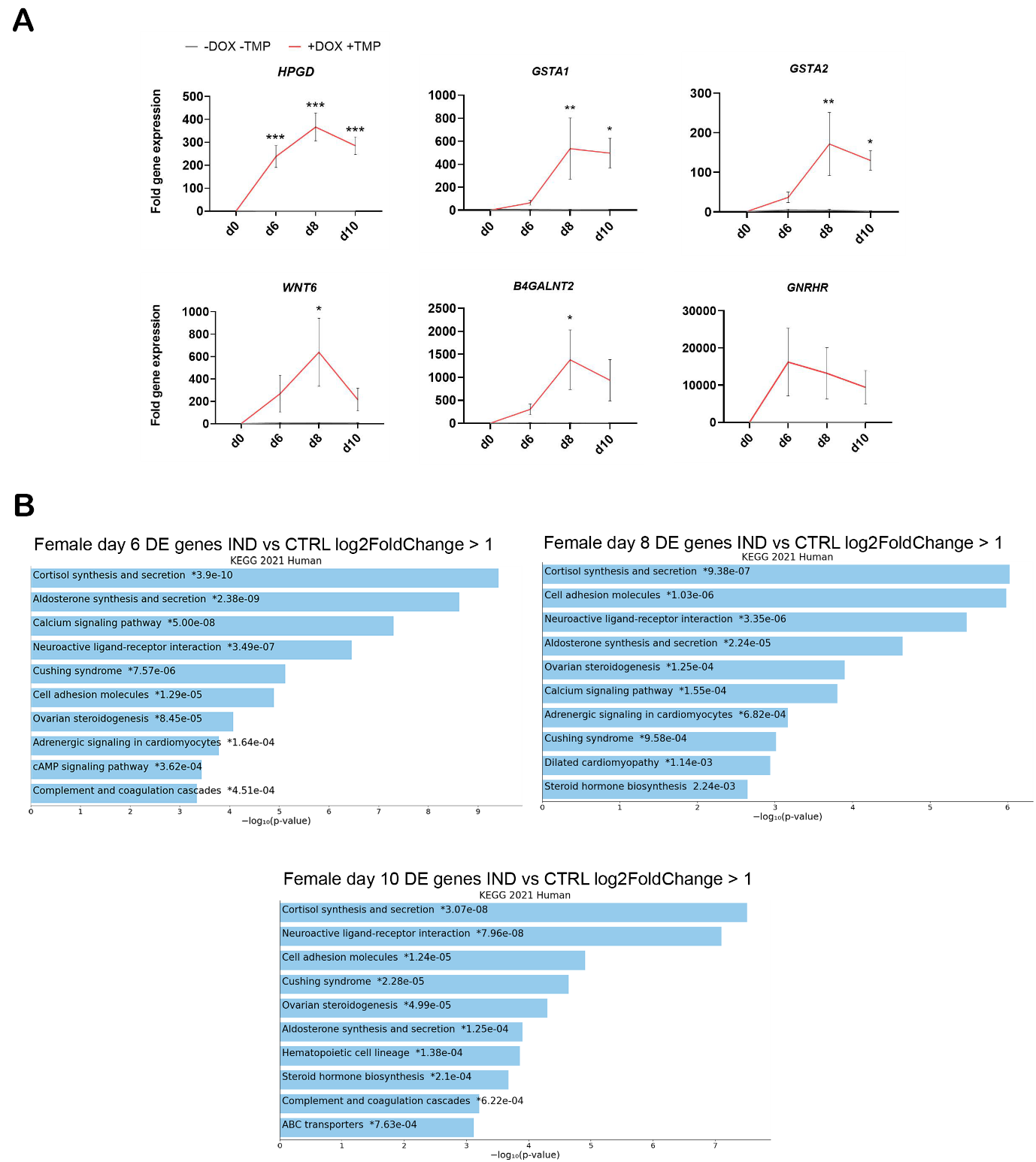


**Supplementary figure 3: Adrenal, testicular, and ovarian steroidogenesis markers were upregulated upon *NR5A1* induction.**

Comparison of the expression of RNA-sequenced non-induced (CTRL, -DOX-TMP) and induced (IND, +DOX+TMP) samples. Known adrenal, testicular and ovarian steroidogenic markers were upregulated upon *NR5A1* induction. Each square represents the expression of a specific gene within a technical replicate. The intensity of gene expression is indicated by a colour scale based on row z-scores (red, highest expression levels; blue, lowest expression levels). d, day.


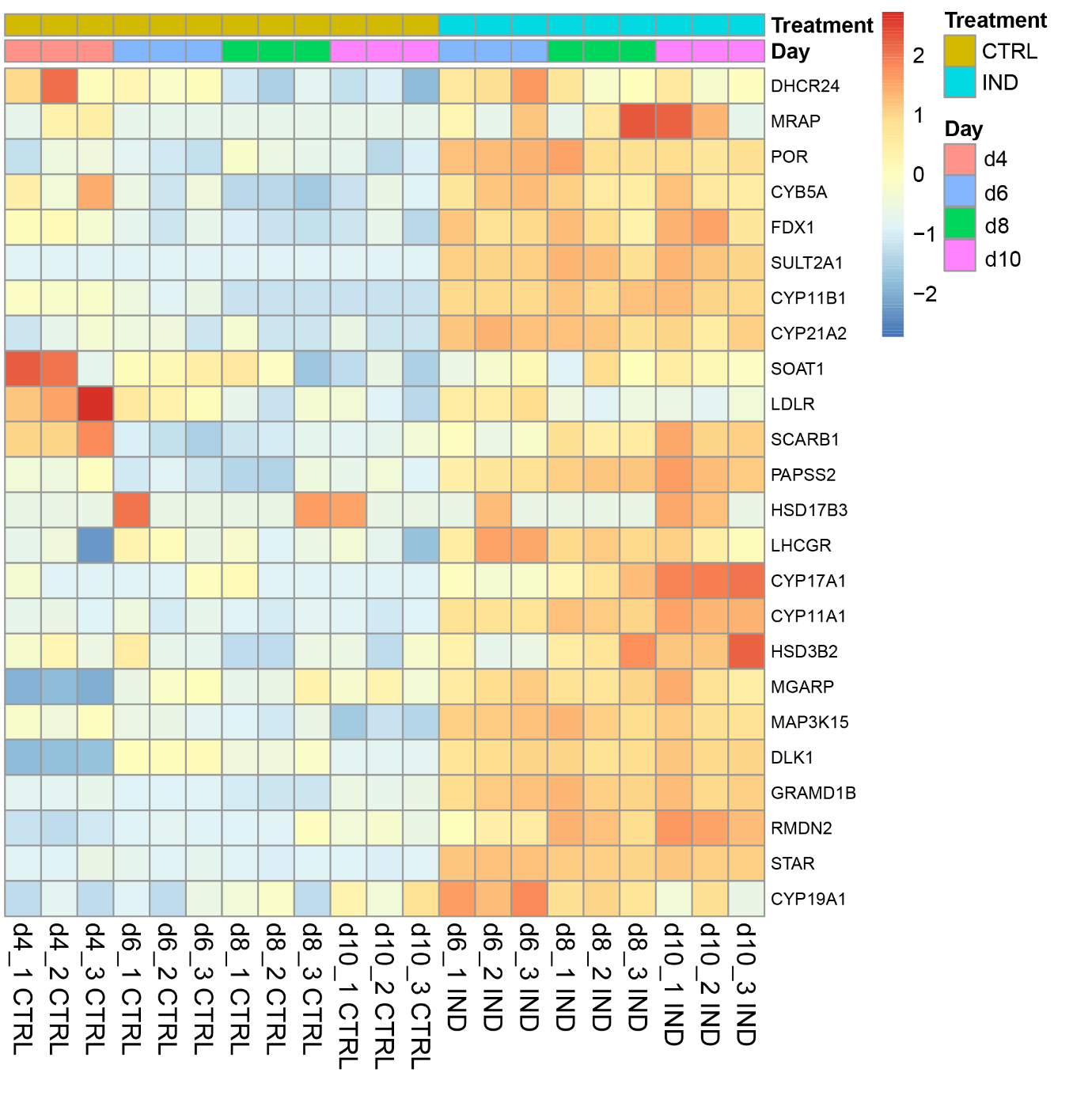

Supplement: Supplementary file 1 — Additional file 1: Supplementary figure 1. Bulk RNA-sequencing analyses showed differences between the control (-DOX-TMP) and induced (+DOX+TMP) conditions. A. Principal component analysis (PCA) comparing samples from different days of differentiation and control (CTRL, -DOX –TMP) and induced (IND, +DOX +TMP) conditions. d; day. B. Flowchart comparing the number of differentially expressed (black), coding (orange), non-coding (blue), upregulated (green) and downregulated (red) genes in induced (+DOX+TMP) and non-induced (-DOX-TMP) conditions at different timepoints in both female and male gonadal differentiation. d; day. Supplementary figure 2. Differentially expressed upregulated genes were mostly associated with (adrenal) steroidogenesis. A. RT-qPCR analysis showed that NR5A1 induction upregulated the markers HPGD, GSTA1, GSTA2, WNT6, B4GALNT2 and GNRHR. Data are reported as mean ± SEM, n=3 biological replicates. Two-way ANOVA; 0.12 (ns), 0.033 (*), 0.002 (**), < 0.001 (***). d; day. B. Bar charts showing the correlated KEGG 2021 human pathways of the female DE genes between control (CTRL, -DOX -TMP) and induced (IND, +DOX +TMP) conditions at days 6, 8 and 10 of gonadal differentiation. The bar charts show the top 10 enriched terms in the chosen library, along with their corresponding p-values. Coloured bars correspond to terms with significant p-values (< 0.05). An asterisk (*) next to a p-value indicates the term also had a significant adjusted p-value (< 0.05). Supplementary figure 3. Adrenal, testicular, and ovarian steroidogenesis markers were upregulated upon NR5A1 induction. Comparison of the expression of RNA-sequenced non-induced (CTRL, -DOX-TMP) and induced (IND, +DOX+TMP) samples. Known adrenal, testicular and ovarian steroidogenic markers were upregulated upon NR5A1 induction. Each square represents the expression of a specific gene within a technical replicate. The intensity of gene expression is indicated by a colour scale based on row z-scores (red, [file 13048_2023_1264_MOESM1_ESM.docx]
